# Aptamers—Diagnostic and Therapeutic Solution in SARS-CoV-2

**DOI:** 10.3390/ijms23031412

**Published:** 2022-01-26

**Authors:** Tomasz Wandtke, Ewelina Wędrowska, Marcin Szczur, Grzegorz Przybylski, Marek Libura, Piotr Kopiński

**Affiliations:** 1 Department of Lung Diseases, Neoplasms and Tuberculosis, Faculty of Medicine, Nicolaus Copernicus University in Toruń, 85-094 Bydgoszcz, Poland; ewelina.wedrowska@gmail.com (E.W.); gprzybylski27@gmail.com (G.P.); mpkopins@hotmail.com (P.K.); 2 Vitalabo Laboratoria Medyczne Sp. z o.o. Grupa Diagnostyka, Gen. J. Hallera 2E Str., 85-795 Bydgoszcz, Poland; marcin@vitalabo.com.pl; 3 Health Care Center of the Ministry of Interior and Administration Department of Traumatology and Orthopedics, 25 Kronikarza Galla Str., 30-053 Krakow, Poland; mareklibura@icloud.com; 4 Krakow Center for Medical Research and Technology, John Paul II Hospital, 31-202 Krakow, Poland

**Keywords:** aptamers, aptasensors, COVID-19, SARS-CoV

## Abstract

The SARS-CoV-2 virus is currently the most serious challenge to global public health. Its emergence has severely disrupted the functioning of health services and the economic and social situation worldwide. Therefore, new diagnostic and therapeutic tools are urgently needed to allow for the early detection of the SARS-CoV-2 virus and appropriate treatment, which is crucial for the effective control of the COVID-19 disease. The ideal solution seems to be the use of aptamers—short fragments of nucleic acids, DNA or RNA—that can bind selected proteins with high specificity and affinity. They can be used in methods that base the reading of the test result on fluorescence phenomena, chemiluminescence, and electrochemical changes. Exploiting the properties of aptamers will enable the introduction of rapid, sensitive, specific, and low-cost tests for the routine diagnosis of SARS-CoV-2. Aptamers are excellent candidates for the development of point-of-care diagnostic devices and are potential therapeutic tools for the treatment of COVID-19. They can effectively block coronavirus activity in multiple fields by binding viral proteins and acting as carriers of therapeutic substances. In this review, we present recent developments in the design of various types of aptasensors to detect and treat the SARS-CoV-2 infection.

## 1. Introduction

Throughout history, people have encountered many threats—one of them undoubtedly being biological factors, including those that have repeatedly caused massive epidemics that have debilitated humanity. Pathogens with such potential are viruses that have caused at least six local or global epidemics in the last century alone. At this point, the Spanish Flu epidemic (influenza H1N1, 1918) should be mentioned, as well as Severe Acute Respiratory Syndrome (SARS; 2003), the H1N1 epidemic (2009), Ebola (2014), and the Middle East Respiratory Syndrome (MERS; 2012) [1].

On 31 December 2019, the Health Commission of the Hubei province, Wuhan, China, reported the first atypical cases of pneumonia in humans [2]. As later identified, a previously unknown coronavirus was responsible for causing them. On 11 February 2020, the World Health Organization (WHO) proposed that the disease caused by this newly identified infectious agent should be called COVID-19 [3], and the agent causing it was designated as the SARS-CoV-2 coronavirus [4]. The virus spread from Wuhan around the world extremely quickly. On 11 March 2020, because of the spreading threat, the WHO declared a pandemic state for the first time in history [5]. So far (2 January 2022), over 289 million infections have been reported, 5.44 million of which have been fatal [6].

### 1.1. Characteristics of Coronavirus SARS-CoV-2

Careful analysis showed that SARS-CoV-2 was most likely zoonotic and transferred from the host animal to humans. Research indicated that the closest relatives of SARS-CoV-2 were coronaviruses detected in bats and pangolins [7,8].

SARS-CoV-2 is currently a serious challenge to global public health. Its appearance has seriously disrupted not only the functioning of health services on a global scale, but also the world economy and, on the local level, social relations.

#### 1.1.1. Transmission

It has been established that the dominant source of virus transmission is the infected micro aerosol excreted from the respiratory tract of an infected person [9,10]. It has been shown that the rapid spread of the SARS-CoV-2 infection is favored by its effective transmission from person to person and is additionally favored by the extended time that must pass from infection to the first symptoms (during which an infected person can then infect healthy people) and the significant percentage of carriers who do not develop symptoms at all after infection [2,11,12,13,14,15].

Based on the analyses, it was determined that the probability of infection after contact with an infected person is as high as 83% [2]. The Basic Reproductive Number (R0), the parameter determining how many people infected with the virus can infect others on average, is 2.2. However, there are reports that put it as high as 6.47 [16].

#### 1.1.2. Symptoms

It has been proven that, in most cases, 5 to as many as 14 days must lapse from the moment of infection to the onset of infection symptoms. Originally, the SARS-CoV-2 infection was characterized by symptoms such as fever, dyspnea, dry cough, diarrhea, or temporary loss of the sense of smell and taste, and, in more severe cases, complications originating from a cytokine storm [17,18,19,20,21,22]. However, with the emergence of new variants of this virus in the population resulting from its natural predisposition to variability in the occurrence of other symptoms, e.g., sore throat in the case of the Alpha/Delta variant, are currently observed.

Based on the data collected so far, it seems that people with a history of comorbidities and the elderly are prone to a more severe course of COVID-19, as well as a higher probability of dying from this disease [23,24,25]. It has been found that an increased probability of death occurs especially in those who have elevated levels of troponin T, IP-10, MCP-3, and IL-1ra [26,27].

#### 1.1.3. Structure of Genome and Virion

Research on the pathogen contributed to its classification into the beta-coronavirus subfamily [28]. The SARS-CoV and MERS viruses were previously classified into the same subfamily [29,30].

**Figure 1:**
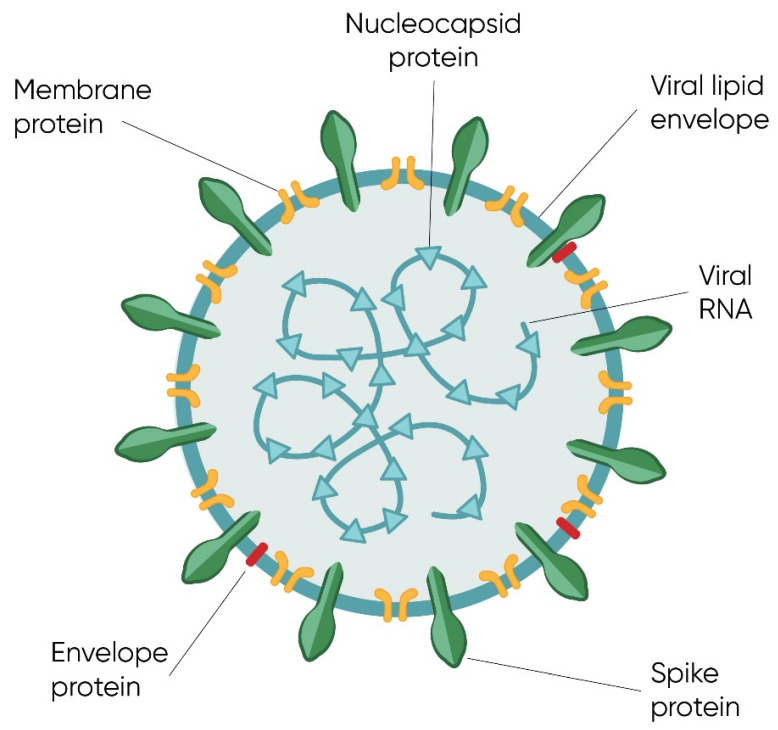
Structure of the SARS-CoV-2 virion. A single particle of the virus is composed of a lipid envelope encompassing structural proteins: S (spike), E (envelope), and M (membrane). Inside the virion, there is viral nucleic acid in a form of RNA, which is surrounded by N (nucleocapsid) proteins.

SARS-CoV-2 was found to have a single-stranded positive RNA genome [29,31,32] of considerable length [33], surrounded by nucleocapsid-forming proteins [31]. It was determined that the similarity of the SARS-CoV-2 virus genome to the SARS-CoV virus genome is significant and amounts to 89.1% [34,35,36]. In the structure of the virion, apart from the nucleocapsid, we can also distinguish a spherical protein–lipid shell [31]. For the graphical presentation of the SARS-CoV-2 virion structure, see Figure 1.

The SARS-CoV-2 genome encodes all the structural proteins of the virus—protein S (spike), E (envelope), M (membrane), and N (nucleocapsid) [34,35,36,37]—as well as the non-structural proteins, including viral RNA-dependent RNA polymerase, replicase–transcriptase complex, RNA helicase [37,38,39,40,41], proteinase, hemagglutinin esterase, and proteins 3a/b and 4a/b play an important role in the virus replication process [41,42]. There are untranslated regions at the 3′ and 5′ ends of the viral genome particle that are involved during the infection cycle in the binding of viral and cellular origin proteins [43].

**Figure 2:**
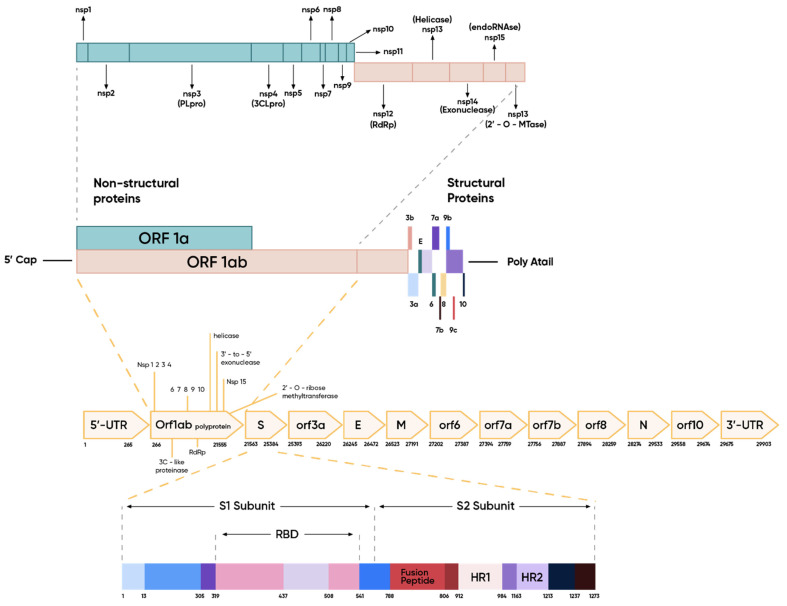
Structure of the SARS-CoV-2 genome (nsp—non-structural protein; RdRp—RNA-dependent RNA polymerase; UTR—untranslated region; ORF—open reading frame; E—envelope protein; M—matrix protein; N—nucleocapsid protein; RBD—region binding domain).

The structure of the SARS-CoV-2 genome is presented in Figure 2.

##### The Spike Protein and Initiation of the Infection Cycle

SARS-CoV-2 has been demonstrated to penetrate the host cells by clathrin-dependent endocytosis [1]. Like its closest relative SARS-CoV, SARS-CoV-2 initiates its infectious cycle by binding to a functional cell surface receptor—human angiotensin-converting enzyme 2 (ACE2) [10,20,44,45,46,47,48,49,50,51,52]. It is found on many different types of cells in the human body but is most abundant on the surface of kidney, intestine, and lung cells, which is where SARS-CoV-2 wreaks the greatest havoc [44,46,47,48,49,50,53].

A leading role in the binding process is played by glycosylated, trimetric, and is anchored in the viral envelope protein S (spike protein) [1,46,47,51,54,55], the proteolytic activation of which takes place under the influence of host enzymes—serine protease and cathepsins [56]. More specifically, the receptor-binding domain (RBD) region of the S protein, located at its C-terminus extending from Arg-319 to Phe-541, is required for host cell receptor binding [1,32,55].

Viral S protein is currently the most frequently chosen target of vaccines, potential drugs, and diagnostic methods, as it determines the cell specificity of the virus, the spectrum of organisms that can be infected by the virus, as well as mortality [32,54,57,58]. This protein exhibits the characteristics of class I viral fusion peptides [54].

**Figure 3:**
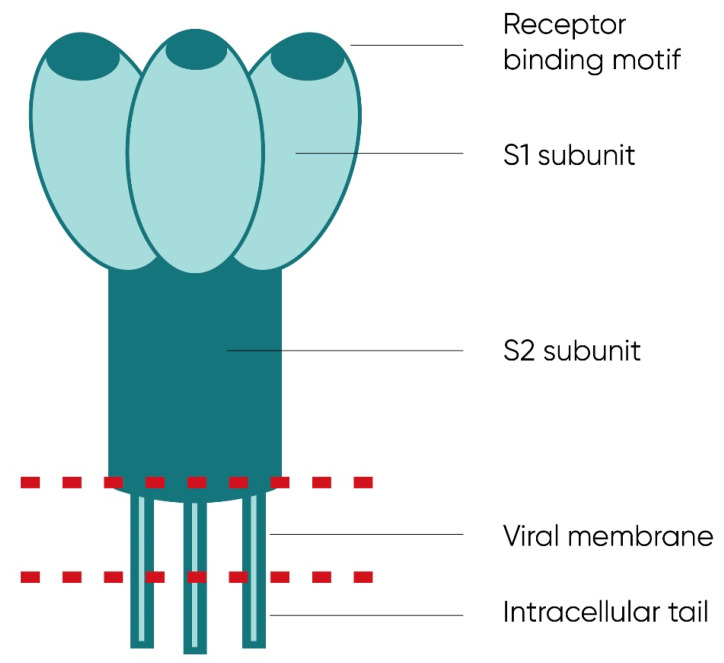
The structure of the SARS-CoV-2 S protein. It is a transmembrane protein composed of two subunits—S1 and S2—located on the outer side of the viral envelope, as well as transmembrane and inner fragments. The S1 protein subunit shows a trimeric structure and presence of the RBD (receptor-binding domain) responsible for the binding of the virion to the ACE2 (angiotensin-converting enzyme 2) receptor on the surface of infected cells.

The structure of the S protein is presented in Figure 3.

##### Other Structural Proteins

The M protein is responsible for the interaction of viral particles with heparan sulfate proteoglycans residues located on the surface of the infected cells [1], and the E protein, viroporin, constitutes an ion channel ensuring optimal pH at the appropriate stages of the infection cycle. This ensures protection of the S protein, and, as a consequence, virus particles with retained infectious potential are released outside the cell [59,60].

Finally, the N protein not only protects the viral genome by creating a nucleocapsid, but also supports the process of packaging the viral genome during the infection cycle, supports the transcription and replication of viral RNA, reduces the host’s immune mechanisms, and contributes to stopping the translation processes of mRNA molecules of cellular origin. The C-terminal domain of this protein shows high affinity for single- and double-stranded DNA fragments as well as for single-stranded RNA—this property is undoubtedly the basis for the use of aptamers in detecting and/or inhibiting this protein [61,62,63,64,65].

### 1.2. Aptamers

#### 1.2.1. Characteristics

Aptamers are single-stranded oligonucleotide (20–80 nucleotides), DNA, or RNA molecules, the properties of which make them suitable for both diagnostic and therapeutic purposes [1,66,67,68,69]. Their high specificity, sensitivity, and binding affinity to the target molecule are particularly important properties of aptamers in the cited context [66,67,70,71,72].

Aptamers can bind a wide range of particles—both inorganic and organic particles, including proteins. Aptamers have been shown to also effectively bind whole viral particles and even cells [66,72,73,74,75]. Such action often leads to their inactivation [66,76]. The act of binding becomes possible thanks to the secondary structure that aptamers obtain in the extra- and intracellular environment—they form stems, loops, hairpins, and even G-quadruplexes [70,72].

#### 1.2.2. Advantages over Monoclonal Antibodies

The binding affinity of aptamers to the targets is at least comparable to that observed in the case of monoclonal antibodies [66,67]. The undeniable advantages of aptamers are also their significant biostability (facilitating transport and storage), bioavailability, and low immunogenicity (compared to monoclonal antibodies), as well as their small size (avoiding spatial collisions after binding on the cell/virus surface) [58,66,70,71,77]. Additionally, the biostability of these molecules in vivo can easily be improved through simple chemical modifications that are easily introduced into their structure [70,71]. This is particularly important in the case of RNA aptamers, which, because of the presence of a reactive hydroxyl group at the second carbon atom of ribose, are more unstable than DNA aptamers [76].

Moreover, the mere acquisition of aptamers is a relatively easy task—they are obtained by an in vitro selection process (SELEX; details of the process are presented in our other publication [66]) [66,68,77,78,79]. This process was first designed and used to obtain aptamers in 1990 [80]. Since then, it has been subjected to continuous improvement and modifications that have an impact on the properties of aptamers (their specificity, cost, and production time) [81]. Importantly, the ease of their production means that it is also possible to obtain aptamers for targets—e.g., toxins—for which the production of monoclonal antibodies is impossible or is significantly impeded, owing to the need to immunize laboratory animals. Other significant advantages are the relatively low cost of such synthesis—it is estimated that, compared to the cost of obtaining monoclonal antibodies, it is 10–50 times lower [66,70,71,82,83,84]—as well as the ability of aptamers to distinguish even very similar targets, e.g., isomers of chemical compounds and point mutations between protein molecules [67,71].

In view of the arguments cited, it is assumed that aptamers can be an extremely inexpensive and effective tool for diagnosis and therapy in the case of the SARS-CoV-2 virus. Moreover, the usefulness of aptamers in this type of application has already been demonstrated many times—numerous studies have been carried out and have confirmed the potential of aptamers in the diagnosis and treatment of various viral diseases (see Table 1 for information about research conducted in the last 5 years; apart from this, we also encourage you to read our previous work from 2015, which was, at that time, a comprehensive review of the achievements in the field of using aptamers to diagnose and treat viral diseases) [66].

## 2. Diagnostics Tools in SARS-CoV-2

Early and correct diagnosis is crucial in the case of people infected with viruses, including, in particular, the SARS-CoV-2 virus. On the one hand, it helps to protect the patient from the severe complications of COVID-19, on the other hand, it allows for the isolation of the patient and thus cuts the chain of infection. In order to make such a diagnosis, it is necessary to have a quick, simple, and repeatable diagnostic method.

Virological diagnostics used nowadays cover a wide range of methods, starting with traditional methods such as cell culture [113], the complement fixation test [114], hemagglutination test [115], electron microscopy [116], moving on to immunological methods determining the level of antiviral antibodies in the patient’s serum, such as immunoenzymatic tests (EIA) and radioimmunoassay tests (RIA) [117], to advanced methods based on the use of a polymerase chain reaction and the amplification of viral nucleic acid, which makes it possible not only to detect the presence of the virus, but, with the use of appropriate techniques, also to enable quantification [69,118,119].

### 2.1. Conventional Diagnostics Methods for the SARS-CoV-2 Infection

#### 2.1.1. Polymerase Chain Reaction

The basic method currently used in the diagnosis of SARS-CoV-2 virus infections is the real-time reverse transcription–polymerase chain reaction (real-time RT-PCR) [1,120,121,122,123,124,125]. The use of the method became possible after the sequence of the virus genome was made public on 7 January 2020, after its earlier isolation from the bronchoalveolar lavage fluid of patients suffering from COVID-19 [126].

This technique is characterized by high specificity in the detection of the viral genome but nevertheless also has some limitations, namely, preparation of the material for testing is a relatively difficult, long, and critical step in the procedure, which does not allow the method to be used in the field. In addition, its use is expensive (the need to use expensive equipment equipped with an optical system, a fluorescence excitation source and its detector), and it also carries the risk of obtaining false positive results (in the case of people who have already fought off the infection) and false negative results (40–60% according to some sources, because of the insufficient sensitivity of the method; material collected from the nasal cavity in some cases does not contain enough genetic material of the virus) [119,121,127,128,129,130,131,132,133,134]. Moreover, the application of the described method requires qualified personnel, and it is therefore rarely possible on-site in the doctor’s office [121,128,131,135].

In the case of the discussed method, the diagnostic material is also important. So far, the presence of SARS-CoV-2 viral particles has been identified in the contents of the nasal cavity, sputum, saliva, and feces of infected persons [136]. Nevertheless, not all patient material is sufficient for diagnostic purposes. For example, it has been proven that saliva—a readily available substance, the collection of which is not invasive and unpleasant for the patient—contains too few virus particles for diagnostics with real-time RT-PCR [1,137]. Therefore, the standard material used to perform a diagnostic test with this method is a nasopharyngeal swab, the collection of which may not be significantly invasive and generally provides detectable amounts of the virus in the event of an infection; however, it can be very unpleasant for the patient.

#### 2.1.2. Serological Tests

Serological tests, especially immunoenzymatic ELISA tests, immunochromatographic strip tests, column chromatography, as well as the use of immunosensors, the principle of which is based on the use of monoclonal antibodies directed against microbial elements or substances that they produce, are slightly less frequently used alternatives to real-time RT-PCR [138,139].

In the case of the SARS-CoV-2 virus, monoclonal antibodies are used to detect antibodies produced by the host organism, most often directed against the S and N proteins of the virus. Depending on the manufacturer, the test uses plasma or serum obtained after centrifuging the patient’s blood [127,140,141,142,143]. Rapid immunochromatographic tests for diagnosis on the spot in the doctor’s office work similarly—the manner in which they work is also based on the use of monoclonal antibodies that detect antiviral antibodies in the patient’s body. The most commonly used diagnostic material in their case is whole blood. However, they allow only a qualitative analysis of the occurrence of antibodies without giving any information about the scale of their production [144].

Unfortunately, the main disadvantage for these types of diagnostic solutions is the long period of time that must elapse from infection with the SARS-CoV-2 virus until the patient’s body produces antibodies. First, about 3 days after the infection, IgM antibodies appear in the patient’s blood, and then after about 2 consecutive days, the first IgG antibodies appear [142,143]. It should also be remembered that antibodies remain in the patient’s blood long after the infection and may contribute to obtaining results that do not correspond to the actual condition of the patient. In fact, they often have greater historical value [143]. Moreover, cross-reactions are possible in tests of this type, the occurrence of which is due to the presence of conserved sequences in the genomes of various coronaviruses [133]. In many cases, this makes it impossible to diagnose the sick person efficiently using this type of test. Moreover, the production of monoclonal antibodies, which are a key element of the tests, is a time-consuming and, above all, costly procedure [127,140]. On the other hand, tests of this type are characterized by a satisfactory specificity and sensitivity (although lower than molecular methods), and, with their help, it becomes possible to diagnose infection quickly and efficiently at the doctor’s office, which may be particularly important in the case of, for example, asymptomatic carriers of the SARS-CoV-2 virus [69,128].

Antibody detection tests for SARS-CoV-2 are limited by the delayed humoral immune response, whereas, for a rapid diagnosis of SARS-CoV-2 infection, rapid antigen detection (RAD) tests for the qualitative determination of the SARS-CoV-2 antigen are applicable. RAD tests detect the viral antigen by coating the device with the SARS-CoV-2 antibody. RAD test results can be interpreted without specialized equipment and are available within 30 min. However, the accuracy and actual performance of these tests is unknown; according to WHO, RAD tests are not recommended for clinical diagnosis [145,146,147,148].

### 2.2. Aptamers as Diagnostics Tool in SARS-CoV-2

Bearing in mind the limitations of the conventional tests, some scientists see a potentially more efficient and more reliable diagnosis in hardware solutions based on the use of aptamers.

The S and N proteins of the virus can be an ideal target for aptamers. It was previously proven in the case of SARS-CoV that, in the initial phase of infection, lasting no more than 10 days, the detection of the viral genetic material and antibodies is significantly less sensitive than the detection of the viral antigen in the form of the viral N protein—the detection sensitivity of this antigen in the initial stage of infection was assessed as nearly 94% [149,150]. Direct detection of viral antigens in diagnostic material using aptamers could reduce, among others, the need to perform an expensive and complicated diagnostic test such as real-time RT-PCR and eliminate the need to wait for the production of antibodies by the infected host, as is the case with the use of serological tests. Moreover, the sensitivity and specificity of aptamers, allowing them to detect even a small amount of virus particles, could make it possible to use saliva as a diagnostic material (collection would be much more patient-friendly) and even identify people infected with SARS-CoV-2 during the latency period [151,152].

The progress made over the last decade in the field of diagnostic solutions based on the use of aptamers detecting various infectious agents, including viral ones, brings us closer than ever to the development and routine use of biosensors based on their principle of action on these molecules. The SARS-CoV-2 virus pandemic is not insignificant in this regard, fueling this development even more. The ease and simplicity of SELEX—the process of acquiring aptamers—makes the acquisition of aptamers against any infectious agent extremely easy. So far, aptamers directed against numerous viral agents have been obtained [66]. Additionally, in the case of the SARS-CoV-2 virus, information about the first of them appeared in the same year the pandemic was declared [58,153]. Their more common use in diagnostic solutions may be influenced by a wide range of diagnostic methods in which they can be used. It has been proven that they perform extremely well as a detection element in methods based on the phenomena of fluorescence, chemiluminescence, and electrochemical changes [154,155,156,157]. It has also been shown that using methods such as a catalytic molecule, cyclic enzymatic amplification, and rolling-circle amplification can significantly enhance the test signal and thus significantly improve their sensitivity [158,159,160].

#### 2.2.1. Isothermal Detection

**Figure 4:**
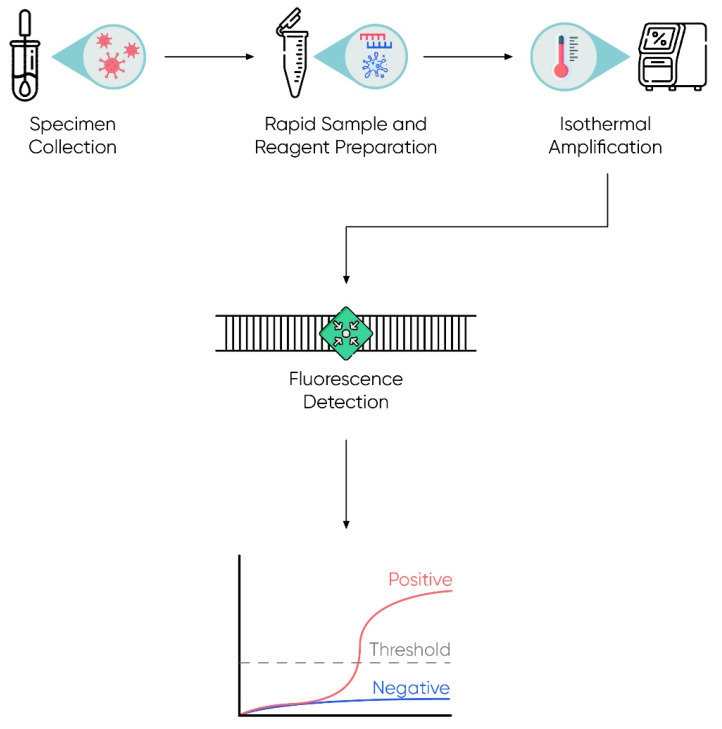
The process of isothermal detection—general view. After the sample is obtained and prepared for testing, isothermal detection is performed. The process may use molecular probes or fluorescence aptamers, and the target of these molecules can be a nucleic acid (RNA or DNA) or a protein, respectively. The signal of the emitted fluorescence is detected by the detector, whose presence proves the occurrence of the molecule in the tested material.

Khan et al. [128] indicated the extremely high usefulness of isothermal techniques in the amplification of nucleic acids and the possibility of their use in diagnostic solutions, including the detection of the SARS-CoV-2 virus. The use of these methods facilitates nucleic acid amplification because they take place at a constant temperature and thus eliminate the need to use classic, expensive thermocyclers that are complicated to operate and used in the PCR reaction. In addition, the authors of the publication indicated that the preparation of material for the study is faster and simpler [128]. The advantage of the presented solution is the fact that the basic substrate of the reaction (detected material) can be both DNA and RNA. Moreover, in the case of the presented technique, it is possible to implement many additional solutions—for example, fluorescently labeled aptamers—which facilitate and simplify the reading of the test result in real time [161,162]. As a result, the use of such a solution significantly reduces the cost of the method, since the aptamer labeling is significantly cheaper than the use of other molecular probes. Moreover, the specificity of the method is almost identical [128]. Such techniques bring us closer to the use of on-site diagnostic instruments in a doctor’s office or at least reduce the time, cost, and complexity of the diagnostic procedure. The subsequent stages of the described method are presented in Figure 4.

One isothermal method that can potentially be used in the diagnosis of the SARS-CoV-2 virus is Nucleic Acid Sequence-Based Amplification. Its advantages can be significantly increased by the implementation of aptamers coupled with a fluorescent dye, as an element enabling easy detection of the presence of a selected nucleic acid in real time [128]. In this respect, Unrau et al. developed the Mango aptamer and showed that it was possible to perform Nested PCR with its use [161,163]. In turn, Engerhart et al. proved that the fluorescent aptamers they constructed allowed for multiplexed detection. At the same time, they confirmed that the reading of the result could take place in the field, since the team developed a way to read it with a cell phone camera [162].

**Figure 5:**
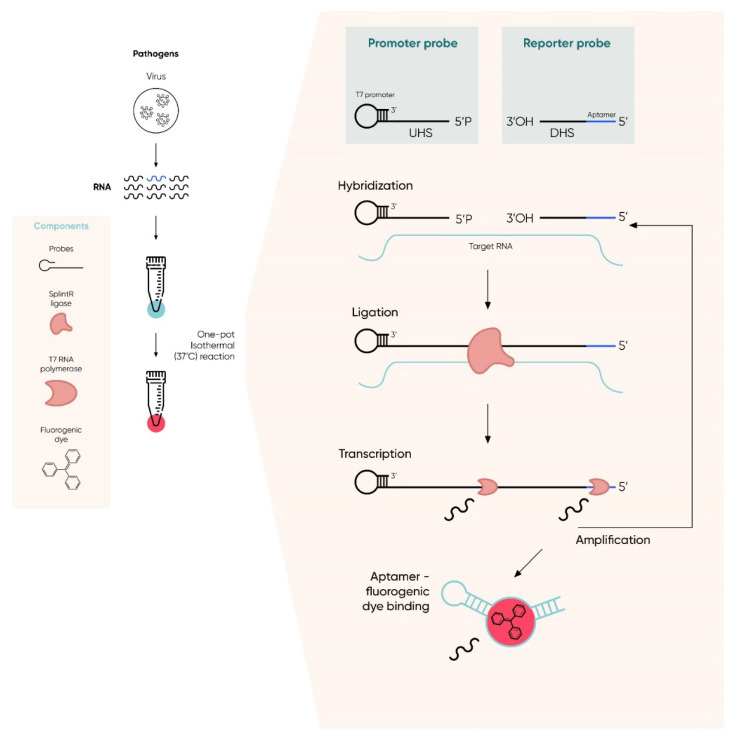
Sensitive splint-based one-pot isothermal RNA detection—process flow. Both reactions take place in one tube. If the tested sample contains the genome (template) of the virus, the promoter and reporter probes will hybridize with it. The SplintR ligase combines the two probes for the first isothermal reaction. Then, T7 RNA polymerase transcribes from a fragment of genetic material that was created by combining both probes forming a second isothermal reaction. The resulting transcript is an aptamer specific to a fluorescent dye—after combining both molecules, fluorescence is emitted that can be detected.

A very interesting achievement in the use of isothermal techniques in the detection of the SARS-CoV-2 virus was made by Woo et al. The team greatly simplified the method technique by making it a simple one-step process (two reactions) in one tube that takes no more than 50 min. The developed method allowed for the detection of the presence of ribonucleic acid, so it perfectly meets the needs of the detection of the SARS-CoV-2 virus genome. The method was named the sensitive splint-based one-pot isothermal RNA detection and consisted of two chemical reactions proceeding at the same temperature: (1) two probes ligation reaction by SplintR ligase and (2) transcription of the ligated probes by T7 RNA polymerase (probes hybridize to template, i.e., viral RNA, if present in the reaction mixture). As a result of both processes, the RNA transcript is in fact an aptamer that exhibits binding affinity for a fluorescent dye and, by binding it, causes its fluorescence. Thus, it appears in the reaction mixture only when an aptamer is formed in it, and the aptamer is formed in it only when one of the reactants is viral nucleic acid (for a graphical presentation of the process, see Figure 5). The team proved the effectiveness of the proposed solution by conducting tests with clinical samples from patients who had previously been infected with the SARS-CoV-2 virus. To obtain a positive test result using the proposed method, a virus RNA concentration not lower than 0.1 aM was sufficient. By analyzing the materials previously diagnosed using the classic qPCR, it was shown that the method proposed by Woo et al. was 85% accurate in the case of materials from infected persons and 100% accurate in the case of materials previously marked as uninfected [164].

#### 2.2.2. Repurposing of SARS-CoV Aptamers

What is worth noting is the fact that, in the initial phase of the pandemic caused by the SARS-CoV-2 virus, effective diagnostic and therapeutic solutions were sought among the repertoire of methods and substances already known.

In earlier years, RNA and DNA aptamers were obtained that effectively recognized the N protein of the SARS-CoV virus, probably the closest relative of the SARS-CoV-2 virus. As proven, they showed a dissociation constant lower than monoclonal antibodies and, additionally, diagnostic solutions (Apt-ELISA, Western blot) based on their use allowed for the identification of the virus [165,166]. It is worth highlighting that the detection threshold of the diagnostic method developed by Ahn et al. was comparable to the detection thresholds of commonly used diagnostic ELISA tests, in which the detection factor is monoclonal antibodies [165].

Chen et al., relying on information about a nearly 90% similarity between the genome of the SARS-CoV virus and SARS-CoV-2, decided to verify whether the previously developed aptamers directed against the viral N protein of the SARS-CoV virus could be adapted to diagnostic solutions aimed at identifying patients infected with the new coronavirus. As has been proven, the sequence similarity of the SARS-CoV-2 virus N protein was sufficient for the aptamers obtained previously to be considered as effective. In the Enzyme-Linked Aptamer Binding Assay test (ELAA), targeting biotinylated aptamers against a substrate-coated viral N antigen, followed by the enzyme horseradish peroxidase (HRP) and its substrate 3.3′,5.5′-teramethylbenzidine (TMB), the ability and selectivity of aptamer binding to the N protein of SARS-CoV-2 was confirmed. Detection was also possible under conditions similar to physiological conditions, i.e., in serum from healthy donors to which viral N protein was previously introduced. The N antigen was detected at a concentration of 10 ng/mL. The authors of the study also proved that the antigen binding efficiency of the aptamer was comparable to the potential observed with commercially available monoclonal antibodies. The analysis of the sequence and structure of the tested aptamers showed that the first two stem-loops were of key importance to their properties—they were responsible for the aptamer’s ability to bind to the viral protein antigen. However, the authors of the study did not analyze the specificity and sensitivity of aptamer binding to the antigen, predicting that its use could significantly accelerate the diagnosis of SARS-CoV-2, since no cases of SARS-CoV infection had been found since 2004, and the similar structure of the N antigen of SARS-CoV-2 to the structure of the N antigens of other coronaviruses infecting humans is minor [133,167].

Using the same aptamers, Jia et al. created an aptasensor to detect the SARS-CoV-2 virus. Its basic structural elements were, apart from RNA and DNA aptamers, optical microfibers coated with graphene oxide. The graphene oxide layer in the proposed solution covered the optical microfibers, ensuring the possibility of immobilizing aptamers on their surface. This is because of the functional groups it introduces. Its presence enables the attachment of more detection molecules—aptamers—which significantly affects the sensitivity of the method. The detection threshold of the purified virus N protein using this solution was remarkably low—it was only 6.25 × 10^−18^ M in the case of using an RNA aptamer as a detection element and 6.25 × 10^−19^ M in the case of using a DNA aptamer. Such a low value of the detection threshold was due to the favorable surface-to-volume ratio, biochemical and optical properties of the graphene oxide forming the sensor surface. In the case of experiments carried out using the N antigen, which was dissolved in fetal bovine serum to imitate the conditions that would be observed in the case of serum collected from patients, the value of the lower detection threshold increased but still oscillated around attractive values for diagnostic solutions dedicated to viral disease detection—in this case, it was 1 × 10^−9^ M. What is worth paying special attention to is the detection time, which was from only 20 s to 3 min with this aptasensor. This is probably the fastest diagnostic solution proposed so far. The authors of the study emphasized the advantages of the proposed solution—the possibility of a qualitative and quantitative assessment, fast detection time, high sensitivity and specificity of the method, low weight, and a simple possibility of miniaturization of the proposed solution [168].

#### 2.2.3. Aptamers Dedicated to SARS-CoV-2

##### Aptamers and Aptasensors

Significant attention in the context of the implementation of diagnostic solutions with the use of aptamers has been devoted to the issue of biosensors, i.e., microdevices including bioreceptors, signal transducers, and an element processing information about the induced signal, allowing for the detection of an infectious agent [169]. A bioreceptor is a detection element in the form of a monoclonal antibody, enzyme, cell, or merely a nucleic acid in the form of an aptamer, the purpose of which is to bind the protein sought, which is most often part of the viral pathogen. In turn, the role of the signal transducer is to convert the event related to the protein binding with the bioreceptor into information that can be read by a laboratory diagnostician [69,169,170,171,172]. Biosensors in which the sensing element is an aptamer are often called aptasensors.

A special opportunity is seen in the use of optical and electrochemical biosensors because their use is simple, eliminates the need for nucleic acid amplification, and thus also involves a significant reduction in costs [173,174,175]. More importantly, the lack of requirements for the preparation of the material before the test means that methods based on the use of optical and electrochemical biosensors can be used on-site, in a doctor’s office, and the test does not require specialized equipment or highly qualified personnel [75,174,175]. A general summary of the advantages and disadvantages of aptasensors is provided in Table 2.

The research of Song et al. was undoubtedly of great importance for the development of dedicated solutions for the detection of the SARS-CoV-2 virus using aptamers, including biosensors. The aforementioned team was the first to obtain two dedicated anti-SARS-CoV-2 aptamers in the ACE2 competition-based SELEX process—designated as CoV2-RBD-1C and CoV2-RBD-4C. They had lengths of 51 and 67 nucleotides, respectively, and a three-dimensional structure of a hairpin. Both bound to different RBD regions of the SARS-CoV-2 protein S and were characterized by very low dissociation constants—5.9 nM for the CoV2-RBD-1C aptamer and 19.9 nM for the CoV2-RBD-4C aptamer—confirming their high binding affinity to the target [58]. The team of Song et al. also confirmed the selectivity of the obtained molecules and their potential usefulness in the case of using material similar to natural diagnostic material—the obtained aptamers were able to bind the dedicated antigen in an 80% plasma solution [58].

Zahashansky and his team [176] developed a solution that significantly reduces the inconvenience for the patient related to the collection of material for examination. Since saliva and not a nasal swab was the basic diagnostic material in their proposed solution, not only were the inconveniences related to the collection of the material important for the patient removed, but the risk for the staff, who were exposed to more frequent and riskier swabs, was also reduced. An important challenge faced by the team of researchers was to achieve a sensitivity of the method that would make it useful in the detection of the virus in saliva, where, as we know, the number of copies of the pathogen is often insufficient for the current gold diagnostic standard for the SARS-CoV-2 virus, which is real-time RT-PCR [176]. The basis of the proposed solution, which made it possible to achieve a sufficiently low detection threshold, was an aptamer previously developed by Song et al.—CoV2-RBD-1C—which acted as a detection element and was directed against the S protein of the virus [58]. Using this aptamer, Zahanshansky et al. created an electrochemical aptasensor in which the aptamer was conjugated to the redox status indicator, methylene blue. The change in the rate of the electron transfer between the redox status indicator and the sensor electrode, which occurs as a result of binding the target to the aptamer and changing the conformation of the latter, could be detected and testify to the presence of SARS-CoV-2 in the tested material. The proposed solution had additional advantages in that it allowed for detection in a relatively short time, i.e., no longer than 30 min, and, most of all, the electrode used for the construction of this biosensor is mass-produced, and its production cost is low—it came from the widely used Shrinky-Dink toy. The team proved that the proposed solution was characterized by a detection sensitivity of the S antigen in saliva at the level of 0.1 fg/mL [176]. Unfortunately, the research was not carried out with diagnostic material from infected patients; therefore, the proposed solution should be subjected to further research.

Another interesting idea was presented by Chen et al. [179]. The research team constructed another biosensor, again using DNA aptamers obtained by Song et al., targeting the S protein of the SARS-CoV-2 virus [58,179]. The detection technique was based on the use of surface-enhanced Raman scattering (SERS), leading to the electromagnetic field enhancement effect, which, according to the authors, significantly improved the sensitivity of the entire solution. In order to construct the test, the team used the aforementioned DNA aptamers conjugated to Cy3–Raman reporter molecules, which were immobilized on the Au nanopopcornsurface. As a result of binding the aptamer to the protein target, it moved away from the sensor surface, and the Raman peak intensity changed in a manner inversely proportional to the concentration of the SARS-CoV-2 virus in the tested sample. As it was proven, the applied solution allowed for the evaluation of SARS-CoV-2 lysates not only qualitatively, but also quantitatively, which was possible in a short time of only 15 min, and its threshold was less than 10 PFU/mL. The biosensor selectivity was also confirmed, since no changes in the reading of the SERS peak intensity were found in the presence of other viruses, including influenza [179].

Pramanik et al. [55] adapted the DNA aptamer obtained by Song et al. against the SARS-CoV-2 protein S into their own solutions. Researchers conjugated the abovementioned aptamer with the dye—rhodamine 6G—and gold nanostars, which made it possible to use the structures and facilitate the detection of the SARS-CoV-2 virus through distance-dependent nanoparticle surface energy transfer spectroscopy. The mechanism of action of the method was based on the suppression of the dye fluorescence when the aptamer was not bound to its specific target and its increase in the opposite situation (binding of the aptamer to the antigen caused its spatial conformation change—straightening—which resulted in an increase in fluorescence resulting from increasing the distance between the gold nanostars and the fluorescent dye Rh-6G conjugated with an aptamer). The team of scientists successfully carried out attempts to detect recombinant SARS-CoV-2 virus spike protein and SARS-CoV-2 spike protein pseudotyped baculovirus using the proposed solution. Detailed analyses showed that the detection time did not exceed 10 min, and its limit for the first experimental model was 130 fg/mL and, for the second one, eight viral particles per 1 mL [55].

An interesting study comparing aptasensors based on different optical detection techniques was carried out by the team of Stanborough et al. [180]. This team benefited from the achievements of Song et al. by using their anti-SARS-CoV-2 DNA aptamer directed against the spike virus protein binding domain [58]. The research allowed the effectiveness of aptasensors in detecting the recombinant S antigen of SARS-CoV-2 virus to be assessed. The compared techniques included surface interferometers, surface plasmon resonance and surface-enhanced Raman spectroscopy. The first two techniques assessed the refractive index, while the third one—surface-enhanced Raman spectroscopy—assessed the vibrational spectrum of the aptamer. The results of the conducted analyses showed that only the last of the abovementioned methods was sensitive enough to be adapted to a possible diagnostic application in the future. The detection threshold in its case reached sub-picomolar values (1 fM) in contrast to the other two techniques, for which the lowest detection thresholds were 250 and 5 nM for surface interferometers and surface plasmon resonance, respectively [180]. The surface-enhanced Raman spectroscopy technique can run with or without marking it, depending on the presence of other particles in the vicinity of the tested particle, the spectra of which may overlap with the spectrum of the element sought in the tested material. In fact, in biological material, the variant with marking is used more often; generally, commercially available Raman-active dyes are used [181,182,183]. In the study, the detection of the SARS-CoV-2 virus antigen by a specific aptamer in the tested material was based on the visual detection of the disappearance of selected spectral peaks in the case of surface-enhanced Raman spectroscopy, which resulted from the decreasing number of -NH groups in favor of bonds formation between the aptamer and the S antigen of SARS-CoV-2. The proposed method was characterized by high sensitivity and specificity, but the process required as much as 2 h of work for the reaction [180]. Moreover, no actual diagnostic tests were used in the course of the study, only a recombinant protein. Further studies are necessary to assess the usefulness of the proposed method when using patients’ sera.

Similarly, the surface-enhanced Raman spectroscopy method was also found to be effective in detecting the new coronavirus by Zavyalova et al. This team also used the COV2-RBD-1C aptamer designed by Song et al. to develop their own diagnostic solution [184]. The vast majority of aptamers obtained so far are directed against selected regions of the SARS-CoV-2 S protein, most often against the RBD of the S1 protein subunit, although information about aptamers directed against other regions of the S protein can also be found, as well as information about aptamers directed against other SARS-CoV-2 proteins. All of them show a diagnostic potential and potentially could be parts of the sensitive aptasensors. Table 3 provides examples (and basic information) of other aptamers with diagnostic potential directed against the S protein of the SARS-CoV-2 virus.

Examples of molecules directed against a protein other than the spike protein are aptamers obtained by Zhang et al. [153]. They proposed their own, different from previous ones, dedicated to the SARS-CoV-2 virus diagnostic solution using aptamers as biosensor detection elements. These researchers were the first to obtain four DNA aptamers from SELEX dedicated to and capable of binding the N protein of the SARS-CoV-2 virus. The dissociation constant of the resulting molecules was different; the lowest was 0.49 nM and related to the Np-A48 aptamer with a length of 58 nucleotides, indicating the strong binding affinity of this molecule to the viral antigen. As a consequence, the team of researchers proposed using the obtained aptamers as detection elements in tests where: (a) the course was similar to the sandwich ELISA test and (b) colloidal gold immunochromatographic strips were used. As a result, with the proposed solutions it was possible to detect the N protein of the SARS-CoV-2 virus at picomolar concentrations—the detection limit in the ELISA test in which aptamers were used as a detection element was about 20 pM. Similar conclusions were provided by the test using test strips—the lowest concentration of the SARS-CoV-2 N protein, for which the result could be read with the naked eye, was 1 ng/mL. The reaction time, which is important in the case of diagnostic solutions, was short, at 15 min. The ability to detect the viral antigen was confirmed in various diagnostic materials—sputum, urine, and serum—although the greatest stability of the aptamer–N protein complexes of the SARS-CoV-2 virus was found in two of them, i.e., sputum and serum. According to the authors of the study, the obtained results particularly indicated the possibility of using the obtained aptamers for the construction of rapid diagnostic tests performed in a doctor’s office [153].

## 3. Aptamers in SARS-CoV-2 Infection Therapy

### 3.1. Repurposing of SARS-CoV and Other Aptamers

So far, no substance has been identified that could be used as a drug for the so-called first line in the case of COVID-19. Only an auxiliary effect in the fight against SARS-CoV-2 has been attributed to the RNA-dependent RNA polymerase inhibitor remdesivir, for which the primary indication was the Ebola infection, but its use in COVID-19 patients has shown some benefits. This preparation received official approval for use in the case of COVID-19 [190,191]. Many of the therapeutic approaches currently under investigation consider the use of convalescent plasma, which may contain potentially effective anti-SARS-CoV-2 antibodies. However, their use is associated with some limitations and risks, since they can be immunogenic [191]. Hence, the search for more effective and safer therapeutic solutions is still being conducted—one of them may be aptamers that are not immunogenic.

What is worth considering is the fact that many studies have shown that drugs whose main active substances are nucleic acids have remarkable therapeutic potential, including in the treatment of viral diseases [192], and their main goal is stopping disease progression from the viral infection cycle [193]. Therapeutic methods using nucleic acids as a therapeutic agent include those based on the phenomenon of RNA interference, particularly with the use of siRNA molecules, as well as those that use ribozymes, antisense oligonucleotides, and aptamers [194]. The detailed mechanism of action of these molecules in respiratory viral diseases is varied [193].

The use of aptamers in COVID-19 therapy is based on one of three possibilities: (a) blocking activity of proteins (e.g., of virus origin, including the RBD of protein S and/or protein M and/or protein N of SARS-CoV-2 or the proteins of cellular origin, e.g., ACE2 receptors) by binding them, (b) stimulating signaling pathways by binding cell receptors (e.g., to induce an immune response), and (c) using aptamers as specific carriers of other substances with therapeutic properties in order to deliver them to infected cells [1,195,196,197].

In the case of the first of the aforementioned strategies, aptamers in particular may target the SARS-CoV-2 virus proteins involved in the processes of adherence, penetration, replication, and release from an infected cell.

Similar to the search for effective diagnostic solutions, the use of the already developed anti-SARS-CoV aptamers as potentially effective agents in the treatment of infections caused by the SARS-CoV-2 virus should be considered when looking for pharmaceuticals. At this point, it is worth noting the observations made by Parashar et al. The research team evaluated the therapeutic potential of the aptamers previously obtained by Ahn et al. [165], targeting the N protein of the SARS-CoV virus [198]. The decisive factor behind this attempt was, as in the case of the attempts undertaken to develop diagnostic tools, the high similarity between the N antigens of both viruses—SARS-CoV and SARS-CoV-2. In fact, the N protein of coronaviruses is one of the most conserved elements preserved between the different types of coronaviruses [61]. The research team subjected the anti-N SARS-CoV RNA aptamers obtained by Ahn et al. to further modifications, including motifs that induced their cell internalization. However, as shown by computer analyses (MFold), the introduced modifications allowed only one of the two used RNA aptamers to maintain the proper structure. The authors postulated that the molecule they modified could be an effective therapeutic agent in a direct inhibition of the SARS-CoV-2 N antigen and an efficient carrier, e.g., for siRNA molecules that inhibit the expression of proteins of viral origin. However, the team did not conduct further laboratory tests; therefore, it is necessary to also verify those hypotheses under experimental conditions [198].

Additionally, two more groups—Shum et al. and Jang et al.—received aptamers that were directed against the SARS-CoV virus, more specifically, against the nsP10 protein of the virus [199,200]. This protein exhibits NTPase/Helicase activity and is involved in many stages of the viral infection cycle, including replication or recombination. It has been proven that its blocking with specific aptamers limited the process of unraveling the genetic material [199,200,201]. Therefore, it is worth considering whether the similarity of the gene and protein sequences of both viruses—SARS-CoV and SARS-CoV-2—is also a source of the effectiveness of these particles in limiting the progression of the SARS-CoV-2 virus infection cycle. So far, no one has conducted any analyses in this area.

Weisshoff et al. presented a slightly different approach to adapting the already existing solutions. As their subject, counting on the faster progress of possible further work, the team of researchers chose the 15-nucleotide, single-stranded DNA aptamer BC007, a molecule that is currently in phase IIa of clinical trials for indications other than infection caused by the SARS-CoV-2 virus [191,202]. It has been shown that BC007 is able to neutralize pathogenic autoantibodies directed against G-protein coupled receptors appearing in the course of, for example, heart and/or cardiovascular diseases [203,204,205]. In the course of the analyses, Weisshoff et al. identified sequences of the RBD region of the viral spike protein and the viral RNA polymerase that could bind to the BC007 aptamer [191]. However, concern about the ability of the aptamer to bind anti-SARS-CoV-2 antibodies produced by the patient’s body was not without significance, since it is known that aptamer BC007 has the ability to bind pathogenic autoantibodies [202]. As shown by the results of the analyses carried out using nuclear magnetic resonance (NMR), isothermal titration calorimetry (ITC), and circular dichroism spectroscopy (CDS), aptamer BC007 specifically and in clinically relevant concentrations bound to the aforementioned viral proteins and did not bind highly specific antibodies for the SARS-CoV-2 virus [191,202]. Interestingly, in the second phase of their studies, the team of scientists showed that aptamer BC007 is able to bind antibodies with a low level of specificity against the SARS-CoV-2 virus, which, according to the authors of the study, most likely constitute autoantibodies against G-protein coupled receptors, whose presence is observed in patients with long COVID-19 [202]. It cannot be ruled out that the use of aptamer BC007 may not only neutralize the virus, but also help in the treatment of the symptoms of long COVID-19 [191,202]. According to the available data, the safety profile of the tested aptamer assessed in the first phase of clinical trials was extremely satisfactory, which creates a number of opportunities for its use, including, perhaps, in the case of the SARS-CoV-2 virus infection [191,206]. However, the analyses carried out by the team were only laboratory analyses. As with previous studies, the aptamer mode of action and efficacy must be verified, at least in live cell cultures infected with SARS-CoV-2.

### 3.2. SARS-CoV-2 Aptamers

In 2020, Parashar et al. postulated verification of the therapeutic potential of the anti-SARS-CoV-2 aptamers obtained by Song et al. [198]. A clear inhibitory potential of the DNA aptamer obtained by the aforementioned team, which was directed against the S protein of the SARS-CoV-2 virus, was demonstrated by the team of Pramanik et al. a year later [55]. Meticulous analysis showed that the particles obtained by Song et al. efficiently bound to the S antigen of SARS-CoV-2. The binding between the aptamer and the S antigen of the SARS-CoV-2 virus involved thymine at position 41, cytosine at position 53, guanine at position 54, and adenine at position 66 in the aptamer molecule, and glutamine, tyrosine, threonine, and lysine in the RBD of the protein S of the SARS-CoV-2 virus [58]. Since it was known that the same amino acids of the spike protein are involved in the process of virus binding to the ACE2 receptor, it could be presumed that this aptamer could not only facilitate the detection of the virus but also, by inhibiting the entry of the virus into the host cells, show a neutralizing effect. In addition, the K_d_ value of both aptamers obtained by Song et al. against the SARS-CoV-2 spike protein is lower than the K_d_ value of the ACE2 receptor against the SARS-CoV-2 virus S antigen (34.6 nM). This may indicate the ability of the aptamer to compete with the ACE2 receptor for binding to the SARS-CoV-2 virus spike protein [58].

By combining the oligonucleotide obtained by Song et al. from gold nanostars and applying such conjugates to cell cultures of the established HEK293T cell line modified to express the ACE2 receptor on their surface, the team of Pramanik et al. demonstrated a reduction in the spread of the virus in the cell culture. Such observations were made in transmission electron and fluorescence microscopy. The limitation of the virus expansion was due to the blocking of the penetration of SARS-CoV-2 particles into the cells and reached the value of 100% at the concentration of aptamer–gold nanostars conjugates of 100 ng/mL. In addition, the team of researchers speculated that, after the application of the aptamer–gold nanostars complexes, not only do they stop the virus from penetrating into target cells by limiting the binding of the viral S antigen to the ACE2 receptor, but also the conjugate itself has the ability to destroy the virus’s lipid envelope [55].

The team of Liu et al. took a similar path. In the SELEX procedure, the team obtained single-stranded DNA aptamers (Aptamer-1 and Aptamer-2) directed against the RBD of the viral S protein. In vitro, the team assessed the therapeutic potential of this oligonucleotide. It was found that the obtained aptamer was able not only to bind with high affinity to the RBD (K_d_ = 7 nM), but also was found to block the interaction of this domain with the ACE2 receptor, thus potentially protecting against infection. It has also been proven that the observed effect was maintained over a relatively wide temperature spectrum, including no major changes at 37 °C, i.e., the temperature of the human body. This makes it a suitable molecule for in vivo applications. The inhibitory effect was aptamer-dose-dependent and was confirmed by carrying out a conventional microneutralization assay using the actual SARS-CoV-2 virus strain designated as USA-WA1/2020 and the established Vero E6 cell line culture [207].

As in the case of diagnostic solutions, almost all cases of aptamers obtained for therapeutic purposes are directed against the S protein of the virus. This is obviously due to the interaction of the viral S protein with the ACE2 receptor on the surface of the infected cells, which is the first event in the infection cycle when the virus enters the body. It seems the most sensible solution to stop the infection cycle at this stage. The number of aptamers produced against the S protein of SARS-CoV-2 seems to support this line of reasoning. Table 4 presents several examples of aptamers (together with general information about them) with therapeutic potential that are directed against the S protein of the virus, other than those described in the above text.

## 4. Conclusions

As is known, the early detection of the SARS-CoV-2 virus and its appropriate treatment are crucial for the effective control of the COVID-19 disease. At present, there is an urgent need to implement in the routine diagnostics rapid, sensitive, specific, and inexpensive tests for the presence of the SARS-CoV-2 virus and to develop effective therapeutic methods. This is particularly important in light of the increasing number of viral mutations (e.g., the recently discovered Delta or Omicron variants) that have the potential to elude currently developed immune-assisted neutralization strategies. In the era of the pandemic, it is the speed and ease of application of a diagnostic method that is of great importance, as well as the possibility of unquestionably differentiating infections caused by influenza viruses and SARS-CoV-2.

Currently, the gold standard in the diagnosis of SARS-CoV-2 virus infections is the real-time polymerase chain reaction. However, it is robust, time-consuming, and expensive. Potentially effective diagnostic tools are also seen in biosensors, including those based on the use of antisense oligonucleotides (ASOs) or aptamers. However, a better solution seems to be the use of the latter, as confirmed by numerous studies cited in this paper. It is worth noting that the use of aptamers to detect proteins (but not antisense oligonucleotides that detect nucleic acids) is associated with a greater sensitivity of the constructed biosensors because, in a single virus particle, there are significantly more proteins on its surface than copies of the genome inside it (a detailed comparison of the use of biosensors to detect viruses, where their mechanism of action is based on antisense nucleotides and aptamers, is discussed in detail, inter alia, in ref. [211]). Moreover, it should be mentioned that aptamers appear to be a versatile diagnostic and therapeutic tool when it comes to new SARS-CoV-2 variants, as demonstrated by, among others, Villa et al. [212] and Zhang et al. [189]. The dynamic development in the field of molecular sciences has allowed for the use of various solutions in the study of aptamer use in SARS-CoV-2 diagnostics, including isothermal techniques, fluorescent optical biosensors, including SERS and electrochemical ones. The rapid and easy-to-use aptamer-based tests enable virus detection without the use of laboratory tools, minimize costs, and are ideal candidates for the development of point-of-care diagnostic devices.

Moreover, aptamer-based techniques are potentially a good option for treating COVID-19 disease. Aptamers, as non-immunogenic and bioavailable therapeutic tools, can effectively block the activity of the coronavirus in many respects, including as carriers of therapeutic substances. In addition, there are a number of options for continuously improving aptamer-based therapies. Although there are no approved uses of these particles in the control of viral diseases to date, it is worth considering the use of already developed aptamers against SARS-CoV in the future.

## Figures and Tables

**Table 1:** Examples of anti-viral aptamers with diagnostic and therapeutic potential obtained within the last five years (VLPs—virus-like particles; g—glycoprotein; VP—virus protein; RT -reverse transcriptase; NS—non-structural protein; HA—Haemagglutinin).

Virus	Aptamer Name	Nucleic Acid Type	Target	Ref.
**THERAPY OF VIRAL DISEASES**				
HPV	Sc5-c3	RNA	HPV-16 VLPs	[85,86]
HSV	DApt	DNA	gD	[87]
Dengue	n/d	RNA	MTase	[88]
Ebola	VPKS-2; VPKS-5	DNA	VP24	[89]
HIV	148.1–38 m	RNA	RT	[90]
	T30175	DNA	Integrase	[91]
	RBA-14	RNA	Rev	[92]
HBV	H01	DNA	HbSAg	[93]
**DIAGNOSTICS OF VIRAL DISEASES**				
HPV	HPV-07	DNA	HPV-16 VLPs	[94]
HCV	C4	DNA	Core	[95]
	A12, A14, A15	DNA	Core	[96,97]
Zika	n/d	DNA	Capsid	[98]
	2, 10	DNA	NS1	[99]
Dengue	n/d	DNA	DENV	[100]
Norovirus	n/d	DNA	VLPs	[101]
	APTL-1	DNA	Capsid	[102]
	n/d	DNA	Capsid	[103]
Influenza	n/d	DNA	H1N1 virus	[104,105]
	A20	DNA	H1N1 virus	[106]
	V46	DNA	H1N1 HA	[107]
	A22	DNA	H3N2 HA	[108]
	P30-10-16	RNA	H3N2 HA	[109]
Ebola	39SGP1A	RNA	GP	[110]
HIV	AntiTat5	RNA	Tat	[111]
HBV	Aptamer 2-19	DNA	HbEAg	[112]

**Table 2:** General advantages and disadvantages of aptasensors and a list of advantages of some aptasensors cited in the text.

Aptasensor by [Ref.]	Advantages	General Advantages of Aptasensors	General Disadvantages of Aptasensors
Zahanshansky et al. [176]	Saliva as the diagnostic material	Different diagnostic material can be used depending on the type of aptasensor Short detection time High sensitivity and specificity Some of the aptasensor undergoes miniaturization and can be used in a physician’s office No need to perform nucleic acid aplification	RNA aptamers as recognition elements are sensitive to exonuclease degradation [177] Necessity of RNA aptamer modification in order to improve their stability [177] Quenching of fluorophores conjugated with aptamers in optical aptasensors by biological components included in the tested material [177] Split aptamers as detection molecules can be applied in aptasensors only in a closed system [178]
	Collection of saliva is less invasive for the patient and safer for the medical staff		
	Detection time		
	Cost production		
Chen et al. [179]	Sensitivity and selectivity		
	Detection time		
	Qualitatively and quantitatively evaluation		
Pramanik et al. [55]	Detection time		
	Needs only 8 viral particles in 1 mL of diagnostic material		
Stanborough et al. [180]	Detection threshold in sub-picomolar range		
	Sensitivity and specificity		
Zhang et al. [153]	Detection threshold in picomolar range		
	Detection time		
	Diagnostic strip form		
	Different types of diagnostic material—sputum, urine, serum		
	Can be performed in a physician’s office		

**Table 3:** Examples of diagnostics aptamers directed against the S protein of SARS-CoV-2 (n/d—no data).

Virus Variant	Nucleic Acid Type	Target	Aptamer Name	Binding Affinity (K_d_) [nM]	Detection Technique	Limit of Detection	Sens	Spec	Ref.
WILD-TYPE	DNA	S protein (N-terminal domain)	SNAP1	39.32 ± 0.12	Lateral Flow Assay	250 pM; 2.5 × 10^7^ copies/mL^−1^	n/d	n/d	[185]
					ELISA	10 pM; 5 × 10^5^ copies/mL^−1^			
			SNAP3	76.59 ± 0.12	n/d	n/d			
		S1 protein (RBD)	nCoV-S1-Apt1	0.33 ± 0.02	AuNPSs colorimetric assay (serum)	3.13 nM			[186]
	DNA (G-quadruplex)	S protein	S14	21.8	ALISA (swab)	2 nM	91%	98%	[187]
	DNA (hairpin)	S1 protein	MSA1	0.023	Colorimetric sandwich assay (saliva)	400 fM 2.4 × 10^8^ particles/mL^−1^	n/d	n/d	[188]
			MSA5	0.012		n/d			
WILD-TYPE	DNA (dimers)	S protein	DSA1N5	0.12 ± 0.02	CoV-eChip (saliva)	saliva: 1 × 10^3^ particles/mL^−1^ spike protein: 1 fM	81%	100%	[189]
ALPHA				0.29 ± 0.04		saliva: 5 × 10^3^ particles/mL^−1^ spike protein: 2.8 fM			
DELTA				0.48 ± 0.06		spike protein: 3.6 fM	n/d	n/d	

**Table 4:** Examples of therapeutic aptamers directed against the S protein of SARS-CoV-2 (n/d—no data).

Aptamer Name	Nucleic Acid Type	Target	Aptamer Application Method	Inhibitory Effect	K_d_/IC_50_	Ref.
SP6	DNA	S protein (different site than RBD)	Application into ACE2 expressing Vero E6 cell line culture infected with the CoV-2 pseudotyped virus	SP6 concentration-dependent reduction of infection rate	K_d_ = 13.9 ± 0.6 nM	[208]
cb-CoV-2-6C3		S1 protein (RBD)	Application into 293T cell line culture expressing ACE2 infected with the SARS-CoV-2 pseudovirus incubated previously with cb-CoV2-6C3	Reduction in viral RNA amount in the cells by 87.01%	K_d_ = 0.13 nM IC_50_ = 9.68 nM	[209]
nCoV-S1-Apt1			Competitive binding assay of ACE2 protein and anti-S1 IgG with nCoV-S1-Apt1	n/d	K_d_ = 0.1 nM IC_50_ = 80.12 nM	[186]
FANA-R8-9	FANA (2′-fluoro-arabinonucleid acid)	S protein (RBD and the larger domain)	ACE2 ELISA assay	The same aptamer effectiveness as the neutralizing RBD-specific antibodies	K_d_ = 14.4 ± 4.6 nM IC_50_ = 1.30 ± 0.2 µg/mL	[210]

## Data Availability

Not applicable.
